# Does Physical Activity Mediate the Associations between Local-Area Descriptive Norms, Built Environment Walkability, and Glycosylated Hemoglobin?

**DOI:** 10.3390/ijerph14090953

**Published:** 2017-08-23

**Authors:** Suzanne J. Carroll, Theo Niyonsenga, Neil T. Coffee, Anne W. Taylor, Mark Daniel

**Affiliations:** 1 Centre for Research and Action in Public Health, Health Research Institute, University of Canberra, University Drive, Bruce 2617, Australia; theo.niyonsenga@canberra.edu.au (T.N.); neil.coffee@canberra.edu.au (N.T.C.); mark.daniel@canberra.edu.au (M.D.); 2 Spatial Epidemiology & Evaluation Research Group, School of Health Sciences and Centre for Population Health Research, University of South Australia, North Terrace, Adelaide 5001, Australia; 3 Discipline of Medicine, University of Adelaide, North Terrace, Adelaide 5005, Australia; anne.taylor@adelaide.edu.au; 4 Department of Medicine, University of Melbourne, St Vincent’s Hospital, Parkville, Melbourne 3010, Australia

**Keywords:** physical activity, cardiometabolic disease, residential environments, descriptive norms, built environment, walkability, mediation, glycosylated hemoglobin

## Abstract

Associations between local-area residential features and glycosylated hemoglobin (HbA_1c_) may be mediated by individual-level health behaviors. Such indirect effects have rarely been tested. This study assessed whether individual-level self-reported physical activity mediated the influence of local-area descriptive norms and objectively expressed walkability on 10-year change in HbA_1c_. HbA_1c_ was assessed three times for adults in a 10-year population-based biomedical cohort (*n* = 4056). Local-area norms specific to each participant were calculated, aggregating responses from a separate statewide surveillance survey for 1600 m road-network buffers centered on participant addresses (local prevalence of overweight/obesity (body mass index ≥25 kg/m^2^) and physical inactivity (<150 min/week)). Separate latent growth models estimated direct and indirect (through physical activity) effects of local-area exposures on change in HbA_1c_, accounting for spatial clustering and covariates (individual-level age, sex, smoking status, marital status, employment and education, and area-level median household income). HbA_1c_ worsened over time. Local-area norms directly and indirectly predicted worsening HbA_1c_ trajectories. Walkability was directly and indirectly protective of worsening HbA_1c_. Local-area descriptive norms and walkability influence cardiometabolic risk trajectory through individual-level physical activity. Efforts to reduce population cardiometabolic risk should consider the extent of local-area unhealthful behavioral norms and walkability in tailoring strategies to improve physical activity.

## 1. Introduction

In efforts to improve population health relating to chronic disease outcomes, interventions have often targeted individual-level health-related behaviors such as physical activity. It is well understood that regular participation in physical activity is protective against the development of chronic diseases, including cardiometabolic disease [1,2,3]. Although some interventions aiming to improve individual-level physical activity have achieved some levels of positive behavior change, the durability of such changes has been questioned [4]. Moreover, interventions focused on individuals can have limited reach, often including only small groups of individuals. Such strategies do not support widespread improvements in health across populations. Environmental factors at the community or population level offer an alternative or complementary focus beyond individual-level intervention with the potential for broader gains in effectiveness [5]. The distribution of health risk in a population is a function of environmental features including norms, resources, opportunities and other local conditions of living [5,6]. Individual risk is shaped, therefore, by *environmental risk conditions* [7]. Unfavorable risk conditions might correspond to only a small difference in the mean population distribution of individual risk but this often heralds a far greater proportion of high-risk individuals in the tail of the risk distribution [5,6]. Interventions targeting high-risk individuals might benefit some such individuals, yet have little impact on the population distribution of risk. Other individuals will become at-risk until the underlying environmental risk conditions are improved and the risk distribution is favorably shifted [5,6]. The criticism that the targeting of environmental risk conditions will have only a small effect on any given individual, needs to be considered against the likelihood of strong benefit to the overall population [6]. Strategies to improve environmental risk conditions can also complement individual-level interventions so that the durability of resultant behavior change is improved [8,9,10]. It is well established that population gains are greatest when environmental and individual-level interventions are employed together [11].

A growing body of empirical evidence links local residential environmental features with both physical activity behavior and cardiometabolic risks and outcomes [10,12,13,14]. Greater local-area socioeconomic status (SES) has consistently been linked to greater levels of physical activity and lesser cardiometabolic risk [12,15,16]. The bulk of this evidence pertains to physical activity and walking behavior for which review articles indicate mixed to fairly consistent positive associations with environmental features including: access to, and availability and variety of, facilities and destinations (e.g., parks and recreational areas, schools, shops and commercial areas); safety (e.g., crime rate, graffiti, incivilities, traffic safety); aesthetics (e.g., greenness, landscaping, upkeep, cleanliness, rated attractiveness); and walkability factors including land use diversity (approximating destinations), residential or population density, street connectivity, and pedestrian infrastructure (e.g., presence and quality of sidewalks, lighting, shade, street furniture) [10,13,17,18,19,20,21,22,23]. Fewer reviews have assessed the evidence for associations between environmental features and cardiometabolic risks and outcomes, such reviews concluded there were reasonably consistent inverse associations between cardiometabolic risk and residential greenspace and walkable urban form including low street intersection and residential densities [12,14,24,25].

Reasonably consistent correlational studies supporting relationships between environmental features and cardiometabolic risk provide a foundation for assessing the potentially causal impact of environments on health [26,27]. Attention to temporality and biological plausibility is needed [26,27]. The majority of studies evaluating place and health relationships have been cross-sectional in design with causal inference precluded by the threat of bi-directionality in the observed associations [12,16]. Longitudinal studies are required to determine the causal direction of relationships with the exposure occurring prior to outcome [26,27].

Biological plausibility requires explanation of the mechanism linking environmental features to cardiometabolic risk [27]. Environmental features may influence cardiometabolic risk through indirect pathways such as individual-level health-related behaviors (e.g., physical activity and diet) [7,12,25]. For example, walkable urban form may enable greater individual physical activity levels and thus improve cardiometabolic health. This mediation premise underlies much research concerning associations between residential built environment features and cardiometabolic risk [28,29,30,31]. Empirical testing of these potential indirect effects is essential to provide evidence on biological plausibility, yet few studies have measured and analyzed such links. It is likely that there are additional pathways through which environmental features influence cardiometabolic risk [12]. Built and social environmental features may also contribute to disease development through stress-related pathways [27]. Adverse environmental conditions may increase stress-levels influencing adverse health-related behaviors such as smoking, alcohol intake, physical inactivity and unhealthful diet. Stress may also directly increase cardiometabolic risk through chronic stress-related physiological responses and significant acute stressors at critical times shaping unfavorable epigenetic responses [12,27,32]. Improving our understanding of the scope of explanatory pathways is necessary to assist in the development of targeted intervention strategies. There is a need for studies that test indirect mediating pathways, ideally using longitudinal designs.

Recent research has implicated spatially variable, local descriptive norms as environmental influences on cardiometabolic risk [33,34,35]. Descriptive norms are *what most people do*, as opposed to injunctive norms which are the ‘shared rules of conduct’ informing on *what ought to be done* [36]. Descriptive norms can be expressed for social groups (i.e., subjective descriptive norms) as well as within spatial settings apart from the social connections (or lack thereof) amongst individuals in that setting (i.e., local descriptive norms) [37,38,39]. Operationally, the local-area prevalence of a behavior or trait defines a local descriptive norm [34,35]. The importance of such influences is highlighted by a longitudinal study indicating that the neighborhood prevalence of overweight/obesity predicted normal weight individuals becoming overweight/obese over 13 years of follow up, independent of individual-level factors and neighborhood SES [33]. Two recent reports using the same population cohort as analyzed here found that greater local descriptive norms for overweight/obesity, physical inactivity and poor fruit intake each predicted increasing cardiometabolic risk (glycosylated hemoglobin, HbA_1c_) over 10 years of follow up, accounting for covariates including individual-level factors, local-area built environment features and area-level SES [34,35]. These studies did not assess, however, the mechanism (i.e., individual-level health-related behavior) through which local descriptive norms influence cardiometabolic risk. The aim of the present study was to longitudinally assess for adults an indirect mediating mechanism, through individual-level physical activity behavior, by which local descriptive norms for overweight/obesity and physical inactivity, and built environment walkability influence 10 year change in HbA_1c_.

## 2. Methods

This study used an observational design incorporating data from the North West Adelaide Health Study (NWAHS; *n* = 4056), a population-based biomedical cohort consisting of randomly selected adults aged 18 years and older. The NWAHS includes three waves of data collection, Wave 1 (2000–2003; *n* = 4056), Wave 2 (2005–2006, *n* = 3563) and Wave 3 (2008–2010, *n* = 2871) over 10 years. NWAHS data were spatially joined using a Geographic Information System (GIS) with built and social environmental datasets including a separate, population-based chronic disease and behavioral risk factor survey (the South Australian Monitoring and Surveillance System (SAMSS)) that was used to construct spatially variable local descriptive norms measures.

The current study was part of the Place and Metabolic Syndrome (PAMS) Project which evaluated the role of residential environment features on the evolution of cardiometabolic risk. The PAMS Project received human research ethics approval from the University of South Australia (P029-10 and P030-10), Central Northern Adelaide Health Service (Queen Elizabeth Hospital; Application No. 2010010), and South Australian Department of Health and Ageing (Protocol No. 354/03/2013 and HREC/13/SAH/53). All participants gave their consent for inclusion before they participated in the study.

### 2.1. Study Area

**Figure 1:**
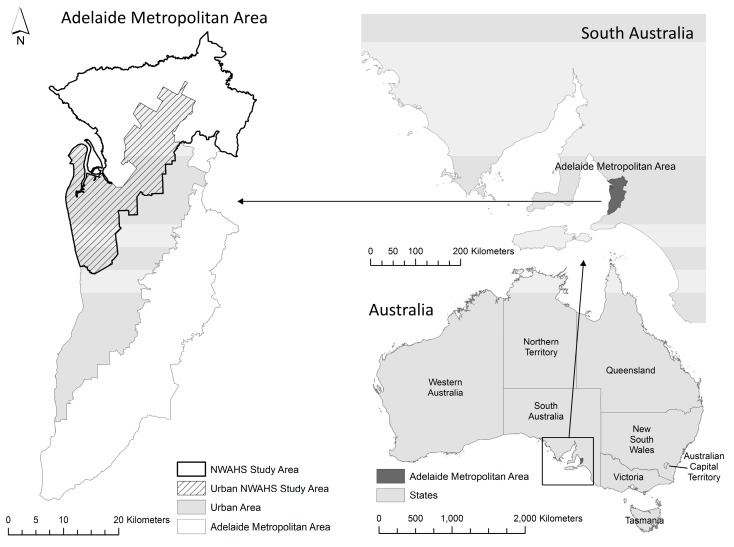
Study area—urban northern and western regions of Adelaide. NWAHS: North West Adelaide Health Study.

At baseline the NWAHS area consisted of the northern and western regions of metropolitan Adelaide (Figure 1), the capital city of South Australia. In 2001, these regions accounted for 38% of Adelaide’s 1.1 million population [40,41]. The northern and western regions of Adelaide were originally targeted for this population study given elevated cardiometabolic risk relative to other Adelaide areas [42,43]. As associations between environments, health behaviors and outcomes can differ between urban and rural regions [44], the study area was limited to urban areas only, defined as Census Collection Districts (CDs) with a population density of >200 persons per hectare [40].

### 2.2. Participants

Individual-level data were drawn from the NWAHS. At each wave of data collection, sociodemographic and behavioral information, and residential address (used to create a participant geo-reference point) were collected using Computer-Assisted Telephone Interviews and self-report paper questionnaires. Biomedical data were collected during clinic visits at each wave, including blood samples to assess HbA_1c_ concentration. Written informed consent was obtained prior to each wave of data collection. Information on data collection and cohort profile has previously been published [45,46].

### 2.3. Measures

#### 2.3.1. Outcome Measure: HbA_1c_

HbA_1c_ concentration (%) was assayed at each data collection wave from fasting blood samples collected at clinic visits. HbA_1c_ reflects 2–3 months time-averaged blood glucose level thus providing a stable marker of glycemic control and related risk [47]. While concentrations of 6.5% or greater are considered indicative of diabetes [48], cardiovascular disease (CVD) risk is considered to rise with HbA_1c_, although the relationship between HbA_1c_ and CVD does not have a defined threshold [49]. Consequently, HbA_1c_ provides an estimate of cardiometabolic risk, that is, risk of diabetes type 2 or CVD, or both.

#### 2.3.2. Individual-Level Physical Activity Information

Individual-level physical activity behavior was defined at Wave 2 as either sedentary (no physical activity reported), some, or meeting recommendations (i.e., ≥150 min of physical activity per week (300 min per fortnight) [50]). Information on time spent participating in walking, moderate and vigorous physical activity was collected using Australian National Health Survey questions as part of the self-reported paper-based questionnaire. Physical activity data were prepared as per recommendations provided for the Active Australia Survey questionnaire [51]. Total time (in minutes) spent participating in physical activity was calculated as:

Time _total_ = time _walking_ + time _moderate_ + 2 × time _vigorous_

This method accounts for the additional health effects of performing vigorous activity as compared to lower intensity levels (i.e., accounts for exercise intensity) [51]. Not all NWAHS participants had complete physical activity data. Where participants had missing physical activity information (i.e., missing information on any of walking, moderate or vigorous activity), total physical activity was coded as missing.

Alternate expressions of physical activity were considered, such as treating physical activity as a continuous variable; however, the zero-inflated distribution with right skew precluded its use as a continuous variable, even after various data transformations were applied. As the operational mediator in the analyses reported here, physical activity was treated as both an independent and dependent variable in mediation models for which it was dummy coded with ‘sedentary’ used as the reference category in the modeling process.

#### 2.3.3. Environmental Measures

Environmental measures analyzed included local descriptive norms for overweight/obesity and physical inactivity, and local-area walkability. Participant-specific environmental exposures were expressed for 1600 m (1 mile) road-network buffers centered on participant residential addresses. The 1600 m buffer represents the distance covered by an average adult walking at a comfortable pace (around 5 km/h) for approximately 20 min [52]. Smaller buffer sizes (1000 m) were considered but dropped due to the small counts of SAMSS survey participants contained within buffers (see below).

Local descriptive norms measures were constructed using geocoded SAMSS data [53]. The SAMSS monitors population trends in chronic diseases and risk factors, using a random sampling of South Australian households identified from the Electronic White Pages telephone directory to recruit participants. Further information on the SAMSS is available elsewhere [53,54]. Geocoded data for adults (18 years and over) were extracted from the SAMSS for the years 2006–2010. These data were aggregated to construct buffer-specific prevalence rates for overweight/obesity (body mass index, BMI ≥ 25 kg/m^2^) and physical inactivity (<150 min of moderate intensity exercise/week) [50,55]. Geocoded SAMSS data were not available prior to 2006 and therefore local descriptive norms could not be temporally matched to the NWAHS at baseline but instead closely approximate Wave 2 exposures in time. Data from 2006 to 2010 were pooled to maximize SAMSS representation within NWAHS participant buffers. To protect confidentiality and support the stability of local descriptive norms estimates, data custodians did not release aggregated norms data for NWAHS buffers with less than 50 SAMSS participants or less than five participants per measurement category. This resulted in sample loss which was substantial at the 1000 m buffer size. Therefore, this unit was removed from further consideration. Further detail on the construction of local descriptive norms is available elsewhere [35].

Walkability was similarly matched to NWAHS data at Wave 2. A walkability index was constructed using data from Adelaide 2007 StreetPro™ road (Pitney Bowes Business Insight, Macquarie Park, NSW, Australia) [56], the 2007 Property Cadastre [57], and the 2007 Retail Database [58]. The walkability index was calculated as the sum of deciles for four components representing connectivity (intersection density) and proximity (dwelling density, land use mix, and net retail area) [59,60,61]. Further detail on walkability components and their construction is available elsewhere [62]. Walkability values range from 4 to 40 with lower values representing less walkable environments. Environmental exposure information was standardized prior to inclusion in analysis models.

#### 2.3.4. Covariates

Analysis models included individual- and area-level covariates, specifically, age, sex, employment status (full-time, part-time, or not in the work force), level of education (university graduate or not), marital status (married/de facto, or single), and smoking status (current smoker, ex-smoker, or never smoked), and area-level income (median weekly household income). Individual-level covariates were selected based on previous empirical research regarding individual predictors of physical activity, cardiometabolic risk and cohort attrition, and analyses of predictors of NWAHS cohort loss to follow-up and missing physical activity data [35,63].

Area-level income was used to represent area-level SES consistent with much of research on area-level SES and health [64]. Area-level income data were extracted from the 2006 Australian Population and Housing Census [65] at the smallest available unit, the CD, and aggregated using the weighted average of values from CDs intersected by the NWAHS participant buffers. Further information on this method is available elsewhere [35]. In 2006, CDs included an average of 220 dwellings [66]. To avoid multicollinearity, only one area-level SES measure was included in the models.

### 2.4. Analyses

Intraclass correlations (ICCs) were calculated from covariance parameter estimates obtained from a multilevel (three-level) empty model (i.e., with no predictors) performed in SAS (version 9.4, SAS Institute Inc., Cary, NC, USA) [67]. These ICCs describe the degree of similarity of HbA_1c_ concentrations for repeated measures within participants and the extent of clustering of participants within suburbs.

The direct and indirect effects of residential exposures on change in HbA_1c_ (the latter through individual-level physical activity) were estimated in Mplus (version 7.4, Muthen & Muthen, Los Angeles, CA, USA) using latent growth models within a structural equation modelling (SEM) approach with Monte Carlo integration [68,69]. The SEM approach enables simultaneous modelling of direct and indirect effects within a single SEM model as opposed to multiple steps. SEM uses a conceptual model with path diagram and a system of linked regression-style equations to capture complex and dynamic relationships within a web of variables in which a dependent variable in one model equation can become an independent variable in other components of the SEM model [68,70,71].

**Figure 2:**
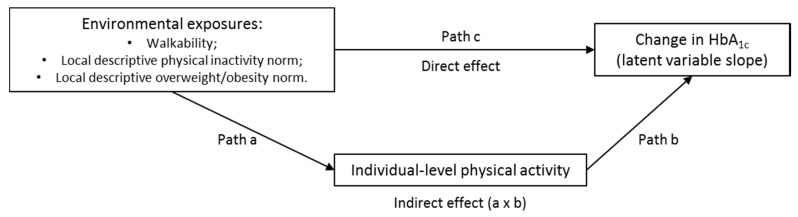
Path model representing hypothesized direct and indirect effects of environmental exposures on change in glycosylated hemoglobin (HbA_1c_).

HbA_1c_ was modeled as a latent variable with a random intercept and slope to allow for participant specific baseline values of HbA_1c_ (intercept), and changes in HbA_1c_ (slope) over time. As shown in Figure 2, each analytic model assessed the influence of an environmental predictor (e.g., local descriptive overweight/obesity norm) on both individual-level physical activity (mediator) and change in HbA_1c_ (slope for latent HbA_1c_ variable), and the influence of individual-level physical activity on change in HbA_1c_. Indirect effects were calculated as the product of coefficients for path a and path b using the model constraint estimation approach [68].

All analysis models included individual-level covariate and area-level income information predicting HbA_1c_ (latent variable intercept and slope) as well as accounting for spatial clustering within suburbs. The use of full information maximum likelihood (FIML) in estimation of effects allowed for the inclusion of cases with missing physical activity data [72,73]. Follow-up sensitivity analyses assessed models using only complete data, with little change in effects noted. Therefore, the results reported here are based on analyses of the broader sample including participants with missing physical activity information. Statistical significance was set at α = 0.05 for all analyses.

## 3. Results

Sample loss due to inclusion criteria is presented in Table 1. There were 2260 participants in the walkability analysis sample, 1926 for the inactivity norm sample, and 1907 for the overweight/obesity norm sample. Characteristics of the individuals in the analysis samples and features of their environments are summarized in Table 2. There were no notable differences with regard to sample characteristics and environmental features between the three datasets. ICC estimates of the similarity of HbA_1c_ concentrations indicated moderate correlation at the individual-level (repeated HbA_1c_ measures over time; ICC = 0.56) and relatively low correlation at the suburb level (ICC = 0.01) consistent with previous reports [74].

Results of mediation models are presented in Table 3. Models estimating the latent variable for HbA_1c_ (i.e., no predictors), indicated average HbA_1c_ of 5.415 (95% CI 5.386 to 5.444, *p* < 0.0001) at baseline, and an increase (worsening) of 0.035 (95% CI 0.029 to 0.040, *p* < 0.0001) in HbA_1c_ concentration (%) per year (not shown in table). In models adjusted for individual-level covariates and area-level income, each of the environmental exposure measures was both directly and indirectly (through physical activity level) statistically significantly associated with change in HbA_1c_. Greater individual physical activity level was negatively associated with change in HbA_1c_ over time in each of the models (point estimates ranging from −0.014 to −0.016, suggesting a ‘protective’ effect).

Greater walkability was negatively associated with change in HbA_1c_ (direct effect, β = −0.008 (95% CI −0.011 to −0.005), *p* < 0.001). Walkability was also positively associated with meeting physical activity recommendations (as opposed to being sedentary; β = 0.035 (0.010 to 0.061), *p* = 0.007), yielding a statistically significant negative indirect effect on change in HbA_1c_ through achieving physical activity recommendations (β × 100 = −0.048 (−0.091 to −0.005), *p* = 0.029). Models indicated a total effect of walkability on change in HbA_1c_ of β × 100 = −0.847 (−1.157 to −0.536; *p* < 0.001) including a total indirect effect of β × 100 = −0.040 (−0.079 to −0.001; *p* = 0.045). Overall, our models show that a one SD increment in walkability (7.45 walkability index points) has a total effect of reducing yearly increases in HbA_1c_ concentration by −0.008 percentage points including a total indirect effect (through physical activity behavior) of reducing the yearly increase in HbA_1c_ by −0.0004 percentage points.

Regarding local descriptive norms, greater local inactivity norm and greater overweight/obesity norm both statistically significantly directly predicted further yearly increases in HbA_1c_ (β = 0.006 (0.001 to 0.011, *p* = 0.015) and β = 0.006 (0.002 to 0.010, *p* = 0.006), respectively). Greater local inactivity norm was negatively associated with meeting physical activity recommendations (compared to being sedentary; β = −0.039 (−0.068 to −0.010), *p* = 0.008). Similarly, greater overweight/obesity norm was negatively associated with meeting physical activity recommendations (β = −0.059 (−0.086 to −0.032), *p* < 0.0001). There were statistically significant indirect effects on change in HbA_1c_ through meeting physical activity recommendations for both local-area norms (physical inactivity norm: β × 100 = 0.061 (0.000 to 0.122), *p* = 0.049; and overweight/obesity norm: β × 100 = 0.085 (0.019 to 0.151), *p* = 0.011). For the local-area inactivity norm, our models indicated a total effect of β × 100 = 0.691 (0.202 to 1.181; *p* = 0.006) with a total indirect effect of β × 100 = 0.070 (0.011 to 0.029; *p* = 0.019) on change in HbA_1c_ concentration. That is, within our models, the total effect of a one SD increment in local physical inactivity norm (7.08% greater physical inactivity prevalence) on the yearly increase in HbA_1c_ concentration was a further rise of 0.0069 HbA_1c_ percentage points per year including an indirect effect of 0.0007 HbA_1c_ percentage points. The total effect of a greater local-area overweight/obesity norm on change in HbA_1c_ was β × 100 = 0.642 (0.239 to 1.046; *p* = 0.002) with a total indirect effect of β × 100 = 0.069 (0.013 to 0.125; *p* = 0.016). A one SD greater prevalence of overweight/obesity (6.18%) was associated with a further yearly rise of 0.006 in HbA_1c_ percentage concentration including an indirect effect of 0.00069 HbA_1c_ percentage points through individual-level physical activity.

## 4. Discussion

The literature reflects a widespread assumption of the premise that individual behavior underlies the multilevel associations between features of residential environments and individual health outcomes. Few studies have actually tested this pathway. This study assessed individual-level health-related behavior as the mechanism by which local-area descriptive norms and built environment walkability shape risk of cardiometabolic disease expressed by 10-year change in HbA_1c_. For our sample, HbA_1c_ increased over time, consistent with previous work indicating well-established links between age and cardiometabolic risk [75,76,77]. Greater individual-level physical activity predicted a reduction in this HbA_1c_ increase over time, similarly consistent with existing knowledge of the inverse relationship between physical activity and cardiometabolic risk [1,2,3]. Compellingly, greater walkability reduced the rate of HbA_1c_ increase over time, while greater local descriptive norms for overweight/obesity and physical inactivity amplified the yearly increase in HbA_1c_. The most novel aspect of this work reflects the test of individual-level physical activity behavior as mediating environmental influences on the evolution of cardiometabolic risk, where physical activity only partially explained the effect of the environmental exposures on change in HbA_1c_. This aligns with the few studies that have previously explicitly tested mediation by physical activity.

The literature contains no previous reports of investigations that tested the indirect mechanism by which local descriptive norms relate to cardiometabolic risk. A small number of studies, however, have assessed the indirect mediating effect of physical activity on the associations between neighborhood walkability and body weight or size. One such study, albeit cross-sectional, of 1200 adult Belgians aged 20–65 years, reported that accelerometer-assessed moderate-to-vigorous physical activity partially mediated associations between greater GIS-derived walkability and greater BMI and waist-to-hip ratio in models adjusted for age, education, work status and neighborhood SES [28]. Similarly, a cross-sectional New Zealand study of 2033 city-dwelling adults aged 20–65 years found that accelerometer-assessed physical activity partially mediated inverse associations between GIS-derived street connectivity and Neighborhood Destination Accessibility Index and body size (BMI and waist circumference) [78]. The study did not, however, observe any associations between body size and land use mix. Thus far, longitudinal evaluations testing physical activity as an indirect mediator of associations between residential walkability and biochemical measures of cardiometabolic risk have not been published. Our study addresses this research gap.

This study, along with the limited number of previous cross-sectional studies on the topic, provides a measure of empirical support of the premise that environmental exposures influence change in cardiometabolic risk through their influence on individual-level health behavior. The mediating effect through physical activity was, however, partial rather than complete, explaining but a small component of the overall estimated direct effects. Partial mediation is similar to the findings of the cross-sectional studies described above. The available evidence thus implies that other mediating pathways may act alongside physical activity behavior in linking environmental exposures to cardiometabolic risk. Other such pathways may include behavioral factors as well as psychosocial and stress-related pathways [7,27]. In the case of local descriptive overweight/obesity norms, individual dietary behavior seems an obvious candidate. However, walkability and local descriptive physical inactivity norms arguably relate specifically to physical activity and sedentary behavior. Sedentary behavior has recently been identified, independent of physical activity, as a risk factor for cardiometabolic disease [79]. Individuals may meet physical activity recommendations while also spending large amounts of daily time sedentary, with consequent adverse health outcomes. Previous research has also documented the clustering of health behaviors [80], and it is possible that such clustering may partially account for findings of a small partial mediation effect for physical activity specifically. Moreover, the features of residential environments can influence health through additional psychosocial pathways, notably chronic stress, whereby allostatic loading yields physiological responses consistent with cardiometabolic risk [7,27]. Further research is needed to investigate such alternative pathways and mediation by multiple clustered factors.

A further consideration is that the mediation effect may be moderated by other factors, both individual-level and area-level. For example, the influence of walkability through physical activity may be moderated by local-area crime and perceived safety. A relatively high walkable area (as opposed to low walkable) that is considered unsafe may have little to no influence on physical activity while the perception of being unsafe may negatively impact cardiometabolic risk through the stress axis. Additional research is needed to elucidate such possibilities. Greater understanding of the pathways linking environments to health outcomes, including under what circumstances and for whom, is essential to inform well-designed, targeted intervention strategies that can account for such inter-relationships.

Despite that fact that this and previous studies found the association between environmental exposures and health was partially rather than fully mediated by physical activity behavior, this does not imply a limited importance of such a mechanism or that it is inconsequential. Nor does it imply that interventions and policy aiming to improve environmental conditions or individual health behaviors are unimportant. The findings of this study regarding the direct effects of walkability on physical activity and cardiometabolic risk concur with previous work in this field. A recent review including a meta-analysis concluded that greater GIS-derived walkability is linked to a greater number of objectively measured (pedometer or accelerometer assessed) daily steps [81]. Other reviews have similarly concluded that built environment walkability, either objectively measured or perceived, is associated with greater levels of physical activity or walking behavior [21,22,25,82]. Regarding walkability and cardiometabolic risk most [12,25,82], but not all [83], reviews have concluded there is fairly consistent evidence of inverse associations between walkability and cardiometabolic risk (most often expressed as body weight).

The current study found, requisite to testing mediation, that local descriptive norms for overweight/obesity and physical inactivity were inversely associated with physical activity and positively associated with change in cardiometabolic risk. This aligns with the results of the few other reported studies of local descriptive norms. One experimental trial reported that individuals who received descriptive norms messages regarding co-workers’ ostensibly greater stair use and walking (i.e., local descriptive physical activity norm) as a result increased their own stair use and walking at the office [84]. A separate study of older adults (average age 87 years, range 80–95 years) in a residential care setting reported that the provision of information about local descriptive norms for physical activity amongst older adults in the general local community (“increasing numbers of exercising adults in the local community”) corresponded to a greater intention to participate in organized physical activities, and greater self-reported participation in physical activity related activities at 3 months of follow-up [85]. Supporting this implication of local context, a longitudinal study (13 years of follow up) of Dutch adults reported greater odds of becoming overweight if they resided in an area with high relative to low neighborhood prevalence of overweight/obesity, in models accounting for age, sex, education and neighborhood deprivation [33]. This same study also reported that individuals were more likely to quit sport if they resided in an area of low sports participation, but this association was not statistically significant in fully adjusted models.

The longitudinal design and analysis reported here is a key strength of this research. The outcome, change in HbA_1c_ was expressed at three time points across 10 years. The temporality of measures supports causal inference [26,27]. Cardiometabolic risk was expressed in a continuous format using a clinical measure, HbA_1c_. Use of a continuous format provides information regarding risk severity, more precisely reflecting the magnitude of change over time. The use of continuous measures has been recommended [77] and such measures provide greater statistical power than categorized measures [86]. The use of a clinically measured outcome is an improvement over using a self-reported measure as is commonly done, as it avoids self-report bias. However, individual-level demographic, smoking and physical activity information (mediator) were self-reported with the consequent potential for self-report bias. Additionally, local descriptive norms measures were aggregated from self-report survey data.

One explanation for an apparent partial rather than full mediating effect of physical activity is the suitability of the means by which it is measured. It is possible that the estimation of mediation effects and a greater proportion of direct effects could be accounted for, if physical activity behavior were operationalized and measured not with the limitations of general self-reported behavioral data, but instead, using an objective measure (e.g., accelerometer) or more specific physical activity measure such as walking for transport. Self-reported physical activity is well known to involve measurement error [87,88]. Local-area walkability, on the other hand, has exhibited more consistent associations with domains of physical activity, notably walking for transport, than general measures of physical activity [10,22,82].

Further strengths of this research include the method of expressing environmental exposures. Walkability was objectively expressed, using a measure previously used in Australia and similar to international standards as applied by the International Physical Activity and Environment Network (IPEN). Local descriptive norms were derived from a separate survey sample, avoiding same-source bias [89]. Environmental exposures were defined within participant-centered road network buffers, per best-practice recommendations [90]. Area-level income was expressed using a method that approximated these buffers. Sensitivity analyses using differently sized road network buffers to represent environmental exposures would have added to this study; however, this was not possible, as noted. Analyses conducted with environmental exposures expressed for differently sized spatial units may yield differences in the nature and strength of relationships found [62,91,92].

Though this study attempted to minimize confounding and reduce bias due to cohort attrition though the inclusion of covariates within analysis models, there remains the possibility of residual confounding from unmeasured influences. Of note, this study did not account for the potential influence of neighborhood self-selection which may have explained some of the association between environmental influences and physical activity and cardiometabolic risk. However, the influence of self-selection is of greater concern in cross-sectional rather than longitudinal studies [93].

## 5. Conclusions

Individual-level health-related behavior represents one potential pathway linking residential environments with cardiometabolic risk; however, this pathway has rarely been empirically assessed. We found that residential walkability, and local descriptive norms for overweight/obesity and physical inactivity were directly and indirectly, through physical activity behavior, associated with change in cardiometabolic risk expressed as HbA_1c_. The indirect pathway through physical activity partially explained the relationship between the environmental exposures and change in cardiometabolic risk, suggesting other mechanisms may be acting in parallel.

This research found environmental exposures predicted both physical activity and change in HbA_1c_, supporting the need to consider local environments in interventions aiming to improve population health. Individual-level intervention strategies need to include an ecological focus, with attention to improving adverse environmental conditions. Future research should investigate additional mechanisms by which environments are related to health outcomes.

## Figures and Tables

**Table 1:** Inclusion criteria and derivation of the three analytic samples.

Criteria	*n*	Reason for Reduced Numbers
NWAHS sample (W1)	4056	-
Geocoded (W1)	4041	15 participants with invalid residential addresses
Residing in urban area (W1)	3887	154 participant addresses outside the urban area
Participated in Wave 2	3362	525 participants did not participate in Wave 2
Did not move (W1 to W2)	2797	565 participants moved between Waves 1 and 2
CVD/diabetes free at Wave 1	2325	472 participants had CVD or Type 2 diabetes at Wave 1
HbA_1c_ data (at least 1 wave)	2324	1 participant lacked at least 1 wave of HbA_1c_ data
Covariate data (W1)	2260	64 participants lacked covariate data at Wave 1
Linked local-area data:		Of participants meeting previous criteria
Walkability	2260	All participants had linked walkability data
Physical inactivity norm	1926	336 participants lacked local physical inactivity norm data
Overweight/obesity norm	1907	353 participants lacked local overweight/obesity norm data

CVD: cardiovascular disease; NWAHS: North West Adelaide Health Study; W1: Wave 1; W2: Wave 2; HbA_1c_: glycosylated hemoglobin.

**Table 2:** Characteristics of individuals (baseline) and environmental features (Wave 2) for the three analysis samples.

Measure	Walkability Sample (*n* = 2260)	Physical Inactivity Norm Sample (*n* = 1926)	Overweight/Obesity Norm Sample (*n* = 1907)
**Individual-Level Characteristics**	**Mean (SD)**	**Mean (SD)**	**Mean (SD)**
Age (years)	50.41 (14.83)	49.97 (15.22)	49.87 (15.18)
Sex (female) *n* (%)	1252 (55.4%)	1061 (55.1%)	1051 (55.1%)
Current smoker *n* (%)	401 (17.7%)	338 (17.6%)	335 (17.6%)
Married/de facto *n* (%)	1476 (65.3%)	1229 (63.8%)	1226 (64.3%)
Education (university graduate) *n* (%)	275 (12.2%)	250 (13.0%)	250 (13.1%)
Not employed *n* (%)	975 (43.1%)	834 (43.2%)	815 (42.7%)
Physical activity-level ^1^:			
*Sedentary n (%)*	553 (43.9%)	461 (32.5%)	454 (32.2%)
*Some n (%)*	449 (26.7%)	381 (26.8%)	381 (27.1%)
*Meets recommendations n (%)*	677 (40.3%)	578 (40.7%)	573 (40.7%)
*Missing n*	581	506	499
HbA_1c_^2^	5.41 (0.45)	5.43 (0.45)	5.43 (0.45)
**Environmental Features**	**Mean (SD)**	**Mean (SD)**	**Mean (SD)**
1600 m buffer area (km^2^) ^3^	3.88 (3.21–4.81)	3.91 (3.31–4.83)	3.91 (3.30–4.84)
Walkability	22.42 (7.45)	-	-
Physical inactivity norm	-	52.76 (7.08)	-
SAMSS *n* per buffer	-	99.4 (32.7)	-
Overweight/obesity norm	-	-	62.85 (6.18)
SAMSS *n* per buffer	-	-	95.6 (31.5)
Area-level income (median weekly household income)	842 (152.78)	835.63 (132.93)	838.45 (131.64)

^1^ physical activity (PA) data less than sample size, proportion expressed using PA sample as denominator; ^2^ 3 NWAHS participants did not have baseline HbA_1c_ data but did have HbA_1c_ data for least one other wave (i.e., sample size reported on is 3 less than in the header); ^3^ median and inter quartile range (IQR) reported. SAMSS: South Australian Monitoring and Surveillance System.

**Table 3:** Direct and indirect effects of environmental exposures on change in HbA_1c_^1^.

Assessed Association	Estimate	95% CI	*p*-Value
**Walkability Models (*n* = 2260) AIC 9646.34; BIC_adj_ 9722.72**			
Walkability predicting ∆HbA_1c_	−0.008	−0.011 to −0.005	0.000
Individual PA predicting ∆HbA_1c_:			
*1. Low (reference = sedentary)*	−0.009	−0.018 to 0.000	0.052
*2. Meets recommendations (reference = sedentary)*	−0.014	−0.021 to −0.006	0.001
Walkability predicting individual PA:			
*1. Low (reference = sedentary)*	−0.009	−0.030 to 0.011	0.347
*2. Meets recommendations (reference = sedentary)*	0.035	0.010 to 0.061	0.007
Indirect effect (×100): through low PA	0.008	−0.012 to 0.029	0.427
Indirect effect (×100): through meets PA recommendations	−0.048	−0.091 to −0.005	0.029
Total indirect effect (×100)	−0.040	−0.079 to 0.001	0.045
Total effect of walkability on ∆HbA_1c_ (×100)	−0.847	−1.157 to −0.536	0.000
**Physical Inactivity Norm Models (*n* = 1926) AIC 8259.17; BIC_adj_ 8330.75**			
Physical inactivity norm predicting ∆HbA_1c_	0.006	0.001 to 0.011	0.015
Individual PA predicting ∆HbA_1c_:			
*1. Low (reference = sedentary)*	−0.011	−0.020 to −0.001	0.039
*2. Meets recommendations (reference = sedentary)*	−0.016	−0.024 to −0.007	0.000
Physical inactivity norm predicting individual PA:			
*1. Low (reference = sedentary)*	−0.008	−0.032 to 0.015	0.490
*2. Meets recommendations (reference = sedentary)*	−0.039	−0.068 to −0.010	0.008
Indirect effect (×100): through low PA	0.009	−0.016 to 0.034	0.496
Indirect effect (×100): through meets PA recommendations	0.061	0.000 to 0.122	0.049
Total indirect effect (×100)	0.070	0.011 to 0.129	0.019
Total effect of physical inactivity norm on ∆HbA_1c_ (×100)	0.691	0.202 to 1.181	0.006
**Overweight/Obesity Norm Models (*n* = 1907) AIC 8151.86; BIC_adj_ 8222.87**			
Overweight/obesity norm predicting ∆HbA_1c_	0.006	0.002 to 0.010	0.006
Individual PA predicting ∆HbA_1c_:			
*1. Low (reference = sedentary)*	−0.011	−0.021 to −0.001	0.028
*2. Meets recommendations (reference = sedentary)*	−0.015	−0.023 to −0.006	0.001
Overweight/obesity norm predicting individual PA:			
*1. Low (reference = sedentary)*	0.015	−0.009 to 0.039	0.219
*2. Meets recommendations (reference = sedentary)*	−0.059	−0.086 to −0.032	0.000
Indirect effect (×100): through low PA	−0.016	−0.048 to 0.016	0.313
Indirect effect (×100): through meets PA recommendations	0.085	0.019 to 0.151	0.011
Total indirect effect (×100)	0.069	0.013 to 0.125	0.016
Total effect of overweight/obesity norm on ∆HbA_1c_ (×100)	0.642	0.239 to 1.046	0.002

^1^: Models adjusted for individual-level age, sex, employment status (full-time, part-time, or not in the work force), level of education (university graduate or not), marital status (married/de facto, or single), and smoking status (current smoker, ex-smoker, or never smoked), and area-level income (median household income). AIC: Akaike’s Information Criterion; BIC_adj_: Sample-size adjusted Bayesian Information Criterion.

## References

[1] Warburton D.E.R., Nicol C.W., Bredin S.S.D. (2006). Health benefits of physical activity: The evidence. Can. Med. Assoc. J..

[2] Thompson P.D., Buchner D., Piña I.L., Balady G.J., Williams M.A., Marcus B.H., Berra K., Blair S.N., Costa F., Franklin B. (2003). Exercise and physical activity in the prevention and treatment of atherosclerotic cardiovascular disease. Arterioscler. Thromb. Vasc. Biol..

[3] Penedo F.J., Dahn J.R. (2005). Exercise and well-being: A review of mental and physical health benefits associated with physical activity. Curr. Opin. Psychiatry.

[4] Greaves C.J., Sheppard K.E., Abraham C., Hardeman W., Roden M., Evans P.H., Schwarz P. (2011). Systematic review of reviews of intervention components associated with increased effectiveness in dietary and physical activity interventions. BMC Public Health.

[5] Daniel M., Lekkas P., Cargo M. (2010). Environments and cardiometabolic diseases in Aboriginal populations. Heart Lung Circ..

[6] Rose G. (1985). Sick individuals and sick populations. Int. J. Epidemiol..

[7] Daniel M., Lekkas P., Cargo M., Stankov I., Brown A. (2011). Environmental risk conditions and pathways to cardiometabolic diseases in Indigenous populations. Annu. Rev. Public Health.

[8] Green L.W., Richard L., Potvin L. (1996). Ecological foundations of health promotion. Am. J. Health Promot..

[9] Green L.W., Kreuter M.W. (2005). Health Program Planning: An Educational and Ecological Approach.

[10] Sallis J.F., Floyd M.F., Rodríguez D.A., Saelens B.E. (2012). Role of built environments in physical activity, obesity, and cardiovascular disease. Circulation.

[11] Daniel M., Green L.W., Breslow L. (2002). Health promotion and education. Encyclopedia of Public Health.

[12] Leal C., Chaix B. (2011). The influence of geographic life environments on cardiometabolic risk factors: A systematic review, a methodological assessment and a research agenda. Obes. Rev..

[13] Kaczynski A.T., Henderson K.A. (2007). Environmental correlates of physical activity: A review of evidence about parks and recreation. Leis. Sci..

[14] Gascon M., Triguero-Mas M., Martínez D., Dadvand P., Rojas-Rueda D., Plasència A., Nieuwenhuijsen M.J. (2016). Residential green spaces and mortality: A systematic review. Environ. Int..

[15] Riva M., Gauvin L., Barnett T.A. (2007). Toward the next generation of research into small area effects on health: A synthesis of multilevel investigations published since July 1998. J. Epidemiol. Community Health.

[16] Diez Roux A.V., Mair C. (2010). Neighborhoods and health. Ann. N. Y. Acad. Sci..

[17] Bauman A.E., Reis R.S., Sallis J.F., Wells J.C., Loos R.J.F., Martin B.W. (2012). Correlates of physical activity: Why are some people physically active and others not?. Lancet.

[18] Grasser G., Van Dyck D., Titze S., Stronegger W. (2013). Objectively measured walkability and active transport and weight-related outcomes in adults: A systematic review. Int. J. Public Health.

[19] Humpel N., Owen N., Leslie E. (2002). Environmental factors associated with adults’ participation in physical activity: A review. Am. J. Prev. Med..

[20] Koohsari M.J., Badland H., Giles-Corti B. (2013). (Re) Designing the built environment to support physical activity: Bringing public health back into urban design and planning. Cities.

[21] McCormack G.R., Shiell A. (2011). In search of causality: A systematic review of the relationship between the built environment and physical activity among adults. Int. J. Behav. Nutr. Phys. Act..

[22] Saelens B.E., Handy S.L. (2008). Built environment correlates of walking: A review. Med. Sci. Sports Exerc..

[23] Van Cauwenberg J., De Bourdeaudhuij I., De Meester F., Van Dyck D., Salmon J., Clarys P., Deforche B. (2011). Relationship between the physical environment and physical activity in older adults: A systematic review. Health Place.

[24] Lachowycz K., Jones A.P. (2011). Greenspace and obesity: A systematic review of the evidence. Obes. Rev..

[25] Malambo P., Kengne A.P., De Villiers A., Lambert E.V., Puoane T. (2016). Built environment, selected risk factors and major cardiovascular disease outcomes: A systematic review. PLoS ONE.

[26] Hill A.B. (1965). The environment and disease: Association or causation?. Proc. R. Soc. Med..

[27] Daniel M., Moore S., Kestens Y. (2008). Framing the biosocial pathways underlying associations between place and cardiometabolic disease. Health Place.

[28] Van Dyck D., Cerin E., Cardon G., Deforche B., Sallis J.F., Owen N., de Bourdeaudhuij I. (2010). Physical activity as a mediator of the associations between neighborhood walkability and adiposity in Belgian adults. Health Place.

[29] Stafford M., Cummins S., Ellaway A., Sacker A., Wiggins R.D., Macintyre S. (2007). Pathways to obesity: Identifying local, modifiable determinants of physical activity and diet. Soc. Sci. Med..

[30] Frank L.D., Kerr J., Sallis J.F., Miles R., Chapman J. (2008). A hierarchy of sociodemographic and environmental correlates of walking and obesity. Prev. Med..

[31] Santana P., Santos R., Nogueira H. (2009). The link between local environment and obesity: A multilevel analysis in the Lisbon metropolitan area, Portugal. Soc. Sci. Med..

[32] Hertzman C., Boyce T. (2010). How experience gets under the skin to create gradients in developmental health. Annu. Rev. Public Health.

[33] Blok D.J., de Vlas S.J., van Empelen P., Richardus J.H., van Lenthe F.J. (2013). Changes in smoking, sports participation and overweight: Does neighborhood prevalence matter?. Health Place.

[34] Carroll S.J., Paquet C., Howard N.J., Coffee N.T., Adams R.J., Taylor A.W., Niyonsenga T., Daniel M. (2017). Local descriptive body weight and dietary norms, food availability, and 10-year change in glycosylated haemoglobin in an Australian population-based biomedical cohort. BMC Public Health.

[35] Carroll S.J., Paquet C., Howard N.J., Coffee N.T., Taylor A.W., Niyonsenga T., Daniel M. (2016). Local descriptive norms for overweight/obesity and physical inactivity, features of the built environment, and 10-year change in glycosylated haemoglobin in an Australian population-based biomedical cohort. Soc. Sci. Med..

[36] Cialdini R.B., Reno R.R., Kallgren C.A. (1990). A focus theory of normative conduct: Recycling the concept of norms to reduce littering in public places. J. Personal. Soc. Psychol..

[37] Carrus G., Bonnes M., Fornara F., Passafaro P., Tronu G. (2009). Planned behavior and “local” norms: An analysis of the space-based aspects of normative ecological behavior. Cogn. Process..

[38] Fornara F., Carrus G., Passafaro P., Bonnes M. (2011). Distinguishing the sources of normative influence on proenvironmental behaviors: The role of local norms in household waste recycling. Group Process. Interg..

[39] Kormos C., Gifford R., Brown E. (2015). The influence of descriptive social norm information on sustainable transportation behavior: A field experiment. Environ. Behav..

[40] Australian Bureau of Statistics (ABS) (2001). Statistical Geography Volume 2: Census Geographic Areas Australia.

[41] Australian Bureau of Statistics (ABS) (2003). Usual Residents Profile 2001.

[42] Dal Grande E., Taylor A., Hurst B., Kenny B., Catcheside B. (2004). The Health Status of People Living in the South. Australian Divisions of General Practice: South. Australian Monitoring and Surveillance System July 2002–December 2003.

[43] South Australian Department of Health (2005). HOS: Self Reported Prevalence of Obesity in the SA Health Regions.

[44] Wilcox S., Castro C., King A.C., Housemann R., Brownson R.C. (2000). Determinants of leisure time physical activity in rural compared with urban older and ethnically diverse women in the United States. J. Epidemiol. Community Health.

[45] Grant J., Chittleborough C., Taylor A., Dal Grande E., Wilson D., Phillips P., Adams R., Cheek J., Price K., Gill T. (2006). The North West Adelaide Health Study: Detailed methods and baseline segmentation of a cohort for chronic diseases. Epidemiol. Perspect. Innov..

[46] Grant J., Taylor A., Ruffin R., Wilson D., Phillips P., Adams R., Price K. (2009). Cohort profile: The North West Adelaide Health Study (NWAHS). Int. J. Epidemiol..

[47] Bennett C.M., Guo M., Dharmage S.C. (2007). HbA_1c_ as a screening tool for detection of type 2 diabetes: A systematic review. Diabet. Med..

[48] IEC (2009). International Expert Committee Report on the role of the A1c assay in the diagnosis of diabetes. Diabetes Care.

[49] Khaw K.-T., Wareham N., Bingham S., Luben R., Welch A., Day N. (2004). Association of hemoglobin A1c with cardiovascular disease and mortality in adults: The European Prospective Investigation into Cancer in Norfolk. Ann. Intern. Med..

[50] Brown W., Bauman A., Bull F., Burton N.W. (2012). Development of Evidence-Based Physical Activity Recomendations for Adults (18–64 Years).

[51] Australian Institute of Health and Welfare (AIHW) (2003). The Active Australia Survey: A Guide and Manual for Implementation, Analysis and Reporting.

[52] Bohannon R.W. (1997). Comfortable and maximum walking speed of adults aged 20–79 years: Reference values and determinants. Age Ageing.

[53] Population Research and Outcome Studies South Australian Monitoring and Surveillance System (SAMSS). http://health.adelaide.edu.au/pros/data/samss/.

[54] Population Research and Outcome Studies South Australian Monitoring and Surveillance System: Survey Methodology, SAMSS Technical Paper Series No. 1/04, August 2004. http://health.adelaide.edu.au/pros/docs/reports/report_samss_tech_paper.pdf.

[55] World Health Organization (WHO) Global Database on Body Mass Index: An Interactive Surveillance Tool for Monitoring Nutrition Transition. http://apps.who.int/bmi/index.jsp.

[56] Pitney Bowes Business Insight (2007). Adelaide 2007 StreetPro and Roads.

[57] Department of Planning, Transport and Infrastructure (DPTI) (2007). South Australian Property Cadastre.

[58] Department of Planning, Transport and Infrastructure (DPTI) (2007). South Australian Retail Database.

[59] Leslie E., Coffee N., Frank L., Owen N., Bauman A., Hugo G. (2007). Walkability of local communities: Using geographic information systems to objectively assess relevant environmental attributes. Health Place.

[60] Frank L.D., Schmid T.L., Sallis J.F., Chapman J., Saelens B.E. (2005). Linking objectively measured physical activity with objectively measured urban form: Findings from SMARTRAQ. Am. J. Prev. Med..

[61] Coffee N.T. (2005). Constructing An Objective Index of Walkability. Master‘s Thesis.

[62] Coffee N.T., Howard N., Paquet C., Hugo G., Daniel M. (2013). Is walkability associated with a lower cardiometabolic risk?. Health Place.

[63] Carroll S.J. (2017). The Contributions of Compositional and Contextual Features of Local Residential Areas to the Evolution of Cardiometabolic Risk over Ten Years in a Population-Based Biomedical Cohort. Ph.D. Thesis.

[64] Schüle S.A., Bolte G. (2015). Interactive and independent associations between the socioeconomic and objective built environment on the neighbourhood level and individual health: A systematic review of multilevel studies. PLoS ONE.

[65] Australian Bureau of Statistics (ABS) (2006). Basic Community Profile (BCP) DataPack.

[66] Australian Bureau of Statistics (ABS) (2006). Australian Standard Geographic Classification Volume 2: Census Geographic Areas.

[67] West B.T., Welch K.B., Galecki A.T. (2007). Linear Mixed Models: A Practical Guide Using Statistical Software.

[68] Muthen B.O., Muthen L.K., Asparouhov T. (2016). Regression and Mediation Analysis Using Mplus.

[69] Muthen L.K., Muthen B.O. (2017). Mplus User's Guide.

[70] MacKinnon D.P., Fairchild A.J. (2009). Current directions in mediation analysis. Curr. Dir. Psychol. Sci..

[71] Gunzler D., Chen T., Wu P., Zhang H. (2013). Introduction to mediation analysis with structural equation modeling. Shanghai Arch. Psychiatry.

[72] Enders C.K., Bandalos D.L. (2001). The relative performance of full information maximum likelihood estimation for missing data in structural equation models. Struct. Equ. Mod..

[73] Arbuckle J.L. (1996). Full information estimation in the presence of incomplete data. Advanced Structural Equation Modeling: Issues and Techniques.

[74] Ukoumunne O., Gulliford M., Chinn S., Sterne J., Burney P. (1999). Methods for evaluating area-wide and organisation-based interventions in health and health care: A systematic review. Health Technol. Assess..

[75] D’Agostino R.B., Vasan R.S., Pencina M.J., Wolf P.A., Cobain M., Massaro J.M., Kannel W.B. (2008). General cardiovascular risk profile for use in primary care. Circulation.

[76] Leiter L.A., Fitchett D.H., Gilbert R.E., Gupta M., Mancini G.B.J., McFarlane P.A., Ross R., Teoh H., Verma S., Anand S. (2011). Cardiometabolic risk in Canada: A detailed analysis and position paper by the cardiometabolic risk working group. Can. J. Cardiol..

[77] Kahn R., Buse J., Ferrannini E., Stern M. (2005). The metabolic syndrome: Time for a critical appraisal. Diabetes Care.

[78] Oliver M., Witten K., Blakely T., Parker K., Badland H., Schofield G., Ivory V., Pearce J., Mavoa S., Hinckson E. (2015). Neighbourhood built environment associations with body size in adults: Mediating effects of activity and sedentariness in a cross-sectional study of New Zealand adults. BMC Public Health.

[79] Proper K.I., Singh A.S., van Mechelen W., Chinapaw M.J.M. (2011). Sedentary behaviors and health outcomes among adults: A systematic review of prospective studies. Am. J. Prev. Med..

[80] Conry M.C., Morgan K., Curry P., McGee H., Harrington J., Ward M., Shelley E. (2011). The clustering of health behaviours in Ireland and their relationship with mental health, self-rated health and quality of life. BMC Public Health.

[81] Hajna S., Ross N.A., Brazeau A.-S., Bélisle P., Joseph L., Dasgupta K. (2015). Associations between neighbourhood walkability and daily steps in adults: A systematic review and meta-analysis. BMC Public Health.

[82] Lovasi G.S., Grady S., Rundle A. (2012). Steps forward: Review and recommendations for research on walkability, physical activity and cardiovascular health. Public Health Rev..

[83] Durand C.P., Andalib M., Dunton G.F., Wolch J., Pentz M.A. (2011). A systematic review of built environment factors related to physical activity and obesity risk: Implications for smart growth urban planning. Obes. Rev..

[84] Priebe C.S., Spink K.S. (2015). Less sitting and more moving in the office: Using descriptive norm messages to decrease sedentary behavior and increase light physical activity at work. Psychol. Sport Exerc..

[85] Koeneman M.A., Chorus A., Hopman-Rock M., Chinapaw M.J.M. (2017). A novel method to promote physical activity among older adults in residential care: An exploratory field study on implicit social norms. BMC Geriatr..

[86] Ragland D.R. (1992). Dichotomizing continuous outcome variables: Dependence of the magnitude of association and statistical power on the cutpoint. Epidemiology.

[87] Brenner P.S., DeLamater J.D. (2014). Social desirability bias in self-reports of physical activity: Is an exercise identity the culprit?. Soc. Indic. Res..

[88] Prince S.A., Adamo K.B., Hamel M.E., Hardt J., Gorber S.C., Tremblay M. (2008). A comparison of direct versus self-report measures for assessing physical activity in adults: A systematic review. Int. J. Behav. Nutr. Phys. Act..

[89] Diez Roux A.V. (2007). Neighborhoods and health: Where are we and where do we go from here?. Rev. Epidémiol. Santé Publique.

[90] Chaix B., Merlo J., Evans D., Leal C., Havard S. (2009). Neighbourhoods in eco-epidemiologic research: Delimiting personal exposure areas. A response to Riva, Gauvin, Apparicio and Brodeur. Soc. Sci. Med..

[91] Openshaw S. (1984). The Modifiable Areal Unit Problem (Concepts and Techniques in Modern Geography).

[92] Flowerdew R., Manley D.J., Sabel C.E. (2008). Neighbourhood effects on health: Does it matter where you draw the boundaries?. Soc. Sci. Med..

[93] Boone-Heinonen J., Gordon-Larsen P., Guilkey D.K., Jacobs D.R., Popkin B.M. (2011). Environment and physical activity dynamics: The role of residential self-selection. Psychol. Sport Exerc..

